# FRIDA: A Four-Factor Adaptive Screening Tool for Demoralization, Anxiety, Irritability, and Depression in Hospital Patients

**DOI:** 10.3390/jcm14196992

**Published:** 2025-10-02

**Authors:** Martino Belvederi Murri, Angela Muscettola, Michele Specchia, Chiara Montemitro, Luigi Zerbinati, Marco Cruciata, Tommaso Toffanin, Guido Sciavicco, Rosangela Caruso, Federica Sancassiani, Mauro Giovanni Carta, Luigi Grassi, Maria Giulia Nanni

**Affiliations:** 1 Institute of Psychiatry, Department of Neuroscience and Rehabilitation, University of Ferrara, 44121 Ferrara, Italy; martino.belvederimurri@unife.it (M.B.M.); chiara.montemitro@unife.it (C.M.); luigi.zerbinati@unife.it (L.Z.); marco.cruciata@edu.unife.it (M.C.); tommaso.toffanin@unife.it (T.T.); rosangela.caruso@unife.it (R.C.); luigi.grassi@unife.it (L.G.); mariagiulia.nanni@unife.it (M.G.N.); 2 Integrated Department of Mental Health and Pathological Addictions, Local Health Trust of Ferrara, 44124 Ferrara, Italy; michele.specchia@edu.unife.it; 3 Department of Mathematics and Computer Science, University of Ferrara, 44121 Ferrara, Italy; guido.sciavicco@unife.it; 4 Department of Medical Sciences and Public Health, University of Cagliari IT, 09124 Cagliari, Italy; federicasancassiani@yahoo.it (F.S.); maurogcarta@gmail.com (M.G.C.)

**Keywords:** demoralization, anxiety, irritability, depression

## Abstract

**Background**: Demoralization, anxiety, irritability, and depression are common among hospital patients and are associated with poorer outcomes and greater healthcare burden. Early identification is essential, but simultaneous screening across multiple domains is often impractical with questionnaires. Computerized Adaptive Testing (CAT) offers a solution by tailoring item administration, reducing test length while preserving measurement precision. The aim of this study was to develop and validate FRIDA (Four-factor Rapid Interactive Diagnostic Assessment), a freely accessible, web-based CAT for rapid multidimensional screening of psychopathology in hospital patients. **Methods**: We analysed data from 472 medically ill in-patients at a University Hospital. Item calibration was performed using a four-factor graded response model (demoralization, anxiety, irritability, depression) in the mirt package. CAT simulations were run with 1000 virtual respondents to optimize item selection, exposure control, and stopping rules. The best configuration was applied to the real dataset. Criterion validity for demoralization was evaluated against the Diagnostic Criteria for Psychosomatic Research (DCPR). **Results**: The four-factor model showed good fit (CFI = 0.947, RMSEA = 0.080). Factor correlations were moderate to high, with the strongest overlap between demoralization and depression (r = 0.93). In simulations, the CAT required, on average, 7.8 items and recovered trait estimates with high accuracy (r = 0.94–0.97). In real patients, mean test length was 11 items, with accuracy of r = 0.95 across domains. FRIDA demonstrated good criterion validity for demoralization (AUC = 0.816; sensitivity 80%, specificity 67.5%). **Conclusions**: FRIDA is the first freely available, multidimensional CAT for rapid screening of psychopathology in hospital patients. It offers a scalable, efficient, and precise tool for integrating mental health assessment into routine hospital care.

## 1. Introduction

The Computerized Adaptive Test (CAT) may be particularly useful for the setting of Consultation Liaison Psychiatry (CLP), possibly contributing to early identification and management of psychopathology.

Demoralization, anxiety, irritability, and depression are among the most common and disabling dimensions of psychopathology in hospital patients. Prevalence estimates suggest that between a third and one-half of in-patients experience clinically significant symptoms [1,2,3,4,5]. These domains are not independent but highly interrelated—recent views support the possibility that the symptoms may interact and promote reciprocally [6,7,8]. While anxiety and depression primarily reflect mood-related symptoms, demoralization has been conceptualized as a syndrome of loss of meaning, subjective incompetence, and motivational impairment [9]. Despite these differences, these constructs frequently co-occur in hospital patients, show strong empirical correlations, and exert reciprocal influences. Assessing them jointly within a multidimensional framework, therefore, allows for capturing both shared and distinct aspects of emotional distress, providing a more comprehensive and clinically informative evaluation. For instance, demoralization may precede or accompany depressive episodes, yet it can also precede it as a distinct syndrome, particularly in the context of medical illness [1,9,10]. Anxiety and depression frequently co-occur, with comorbidity rates exceeding 50%, complicating both diagnosis and treatment [11]. Hostility and irritability are extremely common but less frequently studied [2,12,13]. These psychopathological domains have great clinical salience, both for individual suffering and clinical course: they have been associated with negative outcomes, such as prolonged hospital stays, heightened distress, impaired communication with staff, and reduced quality of life during hospitalization [1,2,14,15]. Other possible consequences of psychopathology include reduced adherence to treatments, increased complications, longer hospital stays, greater risk of readmission or mortality.

Identification of these conditions is important [16], especially considering that the effectiveness of CLP interventions managing them is well established [17]. Screening procedures have been discussed, particularly considering that the majority of patients suffering from such disorders remain undetected and do not remit spontaneously [16,18,19]. This implies a risk of avoidable suffering, impairing medical recovery and even suicidal behaviour within or outside the hospital [20,21]. The implications of screening procedures, however, should not be over-emphasized: for instance, a case has been made on the length and burden of such procedures, especially when multiple dimensions need to be considered simultaneously [22]. Traditional questionnaires may, in fact, require multiple scales or large item sets, reducing feasibility in hospital workflows. Moreover, the interpretation of scores and transmission of results to clinicians may further increase the overall burden, ultimately undermining their utility for decision making at the bedside.

CAT addresses these limitations. By applying Item Response Theory (IRT), CAT tailors item administration to the individual, reducing test length while maintaining or even improving measurement precision. This strategy consistently enhances patient experience, decreases response burden, and provides more accurate estimates compared with fixed-item questionnaires [23,24,25].

Most applications of CAT in psychiatry have focused on unidimensional constructs such as depression or anxiety, often derived from PROMIS or PHQ frameworks [26,27]. Work on multidimensional symptom systems exists but was often conducted within single disorders (e.g., substance use in [25] or outside hospital populations [28]). The CAT-MH system developed by Gibbons and colleagues integrates several domains (depression, anxiety, PTSD, ADHD, suicidality) through multidimensional item response models, but it is proprietary and not freely available [24]. Compared with CAT-MH, FRIDA was specifically designed for hospital patients and focuses on four domains that are highly prevalent in this setting: demoralization, depression, anxiety, and irritability. The inclusion of demoralization and irritability represents a novel contribution, as these constructs are not routinely covered by existing adaptive systems. Furthermore, FRIDA is fully open access, allowing free clinical and research use.

Our group previously conducted IRT studies of demoralization, although we did not develop a CAT [9,14]. The aim of this study was, therefore, to develop and test FRIDA (Four-factor Rapid Interactive Diagnostic Assessment): a freely accessible, web-based CAT designed for the rapid multidimensional screening of demoralization, anxiety, irritability, and depression in general hospital patients.

## 2. Materials and Methods

### 2.1. Participants

This study uses data from a previous study on demoralization in hospitalized individuals [14]. Medically ill patients were recruited between 2018 and 2020 from the Sant’Anna University Hospital in Ferrara, Italy, using a convenience sampling strategy. Participation was offered to in-patients recruited from a range of medical wards—internal medicine, cardiology, endocrinology, nephrology, gastroenterology, pneumology, rheumatology, and oncology. Inclusion criteria were as follows: (1) age ≥ 18 years; (2) capacity to engage in a clinical interview; (3) absence of delirium or other cognitive impairment; and (4) fluency in Italian. After receiving a full explanation of the study, all participants provided written informed consent; no financial or material compensation was offered. Of the approximately 646 patients invited, 472 agreed to participate (response rate 73%). A trained research assistant then conducted a one-to-one session consisting of a semi-structured interview and a battery of psychometric questionnaires; the session lasted approximately 60 min. The study was approved by the Ethical Committee of the Emilia-Romagna Region, “Central Emilia” area (CE-AVEC).

### 2.2. Measures

Demoralization. Demoralization was quantified in two ways. First, patients completed the twenty-four-item Demoralization Scale (DS-24) [14,29], which asks respondents to indicate how often in the previous fortnight they have experienced feelings such as discouragement, loss of meaning or a sense of personal failure, on a five-point scale ranging from 0 (“never”) to 4 (“all the time”).

The interview-based reference we used was the Demoralization module of the Diagnostic Criteria for Psychosomatic Research (DCPR/D) [30,31,32]. The DCPR diagnoses demoralization when, for at least one month, the patient perceives a failure to meet important expectations, feels unable to cope with pressing problems and reports persistent hopelessness or helplessness [33]. Trained psychologists applied the module during the same session as the questionnaires. In our previous study, the DS-24 showed a clear ability to distinguish clinically defined cases of demoralization from non-cases, with an area under the ROC curve of 0.82 compared with the reference diagnostic interview [14]. Internal consistency in the present series was α = 0.94 (0.93–0.95).

Anxiety. To assess symptoms of anxiety, we used the six-item Anxiety subscale of the Brief Symptom Inventory-18 (BSI-18) [34], which asks respondents to rate how much they have been bothered by each symptom in the past seven days on a five-point Likert scale (0 = Not at all to 4 = Extremely). Internal consistency in the present series was α = 0.85 (0.83–0.87).

Irritability and hostility. To assess irritability and hostility, we selected a set of exploratory items from the BSI-53 based on prior evidence linking these symptoms to demoralization, anxious preoccupation, impaired quality of life, interpersonal conflict, and difficulties in emotional expression (2). These items were included as candidate indicators for a distinct irritability dimension in subsequent dimensional and adaptive modelling. Internal consistency in the present series was α = 0.82 (0.79–0.84).

Depressive symptoms. Depression was assessed with the Italian version [35] of the Patient Health Questionnaire-9 (PHQ-9) [36]. Respondents indicate the frequency of nine DSM-IV depressive symptoms over the previous two weeks on a four-point scale from 0 (“not at all”) to 3 (“nearly every day”). In the wider literature, total scores of 5, 10, 15, and 20 represent cutpoints for mild, moderate, moderately severe and severe depression, respectively. Internal consistency in the present series was α = 0.83 (0.81–0.85).

All instruments were administered using validated Italian versions, ensuring linguistic and cultural appropriateness for the study population.

### 2.3. Statistical Analysis

We used methods based on the Item Response Theory (IRT). IRT allows one to model the non-linear relationship between item responses and underlying latent traits, and provides item-level information such as thresholds and discrimination parameters [37,38].

Item calibration was performed using a multidimensional graded response model (GRM) implemented in the *mirt* R package (version 1.14.3.) [37]. First, we tested separate models for each bank, extracting item information functions. A four-factor structure—reflecting demoralization, anxiety, irritability, and depression—was then adopted, based on theoretical considerations and interpretability. The four-factor graded response model was estimated using full-information item factor analysis with the QMCEM algorithm (5000 quasi–Monte Carlo points, Ramsay acceleration, BFGS M-step optimizer). We checked the IRT model assumption of local independence by inspecting the residual correlation matrix and Q3 statistics with quasi-Monte Carlo integration.

We developed the CAT engine using the *mirtCAT* R package [39], based on the four-factor model. First, we assessed efficiency and accuracy by conducting simulations with 1000 virtual respondents, whose data were artificially generated from the fitted model; then, we applied the best-performing configuration to the real dataset, evaluating its behaviour with the actual patient responses. In particular, we explored different combinations of starting and stopping rules, item selection criteria, and exposure controls. We tested multiple starting rules (random versus adaptive selection from high-information items) and item selection methods suitable for multidimensional models, including random, determinant-based (Drule) and Kullback–Leibler divergence (KL, KLn). Exposure control vectors were used in some conditions to prioritise well-performing and clinically relevant items identified in the calibration phase. Stopping criteria included the following: (1) lower bounds on measurement precision for each latent trait, expressed as a maximum acceptable standard error (0.35–0.50); (2) fixed minimum and maximum test lengths, initially set between 4 and 44 items, and later narrowed based on simulation performance; (3) a maximum change in ability estimates across successive items (Δθ), as an additional stability criterion.

For clinical use, we built a freely accessible custom web-based graphical interface to present instructions, collect demographic information, and administer items via radio-button responses. We planned to present users with a graphical tool to represent theta values as continuum, plus a highlighted warning when theta exceeded the value of 0, corresponding to the midpoint of the standard normal distribution of theta. For demoralization, we were also able to examine this criterion’s validity using the DCPR diagnosis of demoralization as the gold standard. Thus, we examined the sensitivity and specificity of theta = 0 using AUC-ROC analyses, hoping to maximize sensitivity and negative predictive value.

## 3. Results

### 3.1. Sample Characteristics

The sample included 472 hospitalized patients (212 *M* and 260 *F*), with a mean age of 62.9 ± 17.7 years (Supplementary Table S1). Most participants were married or cohabiting (56%) and lived with a spouse or partner (56%), while 26% lived alone. Educational attainment was generally low, with 35% having only primary school and 6.8% reporting no qualifications. The most frequent admission reasons were cardiovascular (18%), gastroenterologic (17%), and neurologic (11%) conditions, and 60% of participants were retired.

### 3.2. Item Response Theory Model

The four-factor model converged within 56 iterations, yielding acceptable fit indices (M2 = 3576, df = 896, RMSEA = 0.080, 90% CI = 0.077–0.082; SRMSR = 0.079; CFI = 0.947; TLI = 0.944). The standardized factor loadings indicated substantial variance accounted for by the factors, with SS loadings of 11.60, 3.70, 3.61, and 4.27 for Factors 1–4, corresponding to proportions of variance of 26.4%, 8.4%, 8.2%, and 9.7%, respectively. Factor correlations were generally moderate to high, the strongest being between Factor 1 (Demoralization) and Factor 4 (Depression; *r* = 0.93), followed by correlations of 0.759 between F2 (Anxiety) and F3 (Hostility). Moderate correlations were observed between F1 and F2 (0.671), F1 and F3 (0.526), and F3 and F4 (0.498).

**Figure 1:**
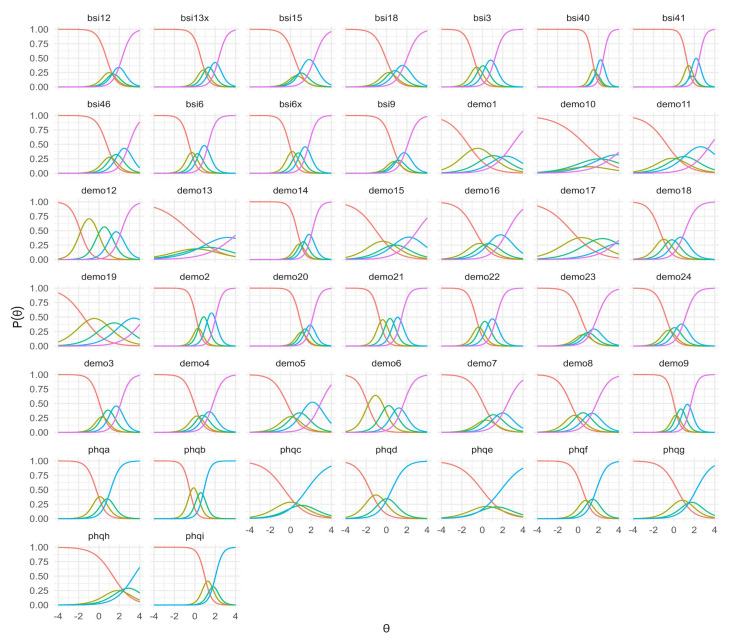
Item Characteristic Curves for all FRIDA items.

The Item Characteristic Curves (ICCs) (Figure 1) show the expected graded response patterns for all items, with ordered category curves and smooth transitions across the latent trait continuum. Most items exhibit well-separated thresholds, suggesting adequate discrimination between adjacent response categories. A few items display category curves with limited peak probability (e.g., extreme categories used infrequently), which may reflect low endorsement rates rather than model misspecification.

Item fit statistics indicated that the majority of items demonstrated adequate fit to the model. A subset of items (e.g., demo2, demo3, demo6, demo9, demo11, demo15, demo16, demo19, demo23, bsi46, phqd) showed significant misfit at *p* < 0.05, though the associated RMSEA.S_X2 values were generally small (≤0.035), suggesting that misfit was modest in magnitude. These results, together with the generally high factor loadings and communalities (Supplementary Table S2), supported the adequacy of the four-factor solution in representing the observed item response patterns.

The local independence assumption was supported by residual pairwise correlations between items below 0.50, except that between phq_g and phq_c (R = 0.508), well below problematic values (≈0.60–0.70). All items were retained in the item bank.

### 3.3. Computerized Adaptive Testing

The CAT was developed from the four-factor model: in its final version, item selection began with demoralization item 18, followed by the determinant-based rule weighted by posterior ability estimates (DPrule) for item selection. Stopping criteria combined trait-specific precision thresholds (maximum standard errors of 0.45 for demoralisation and depression, 0.50 for anxiety and irritability), maximum change in ability estimates between successive items of 0.01 on each factor, and test length limits of 4–25 items. Greater exposure control was given to items with stronger psychometric and information properties identified during calibration.

**Figure 2:**
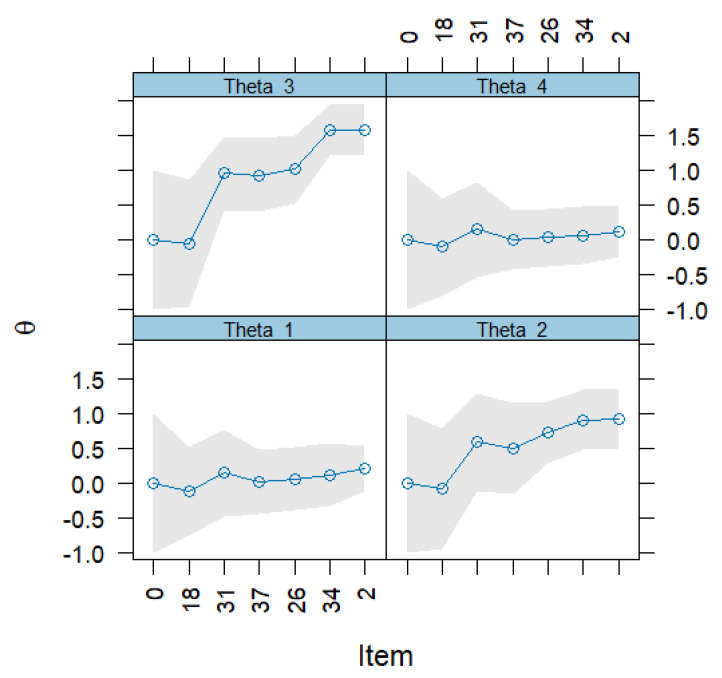
Example of screening with the Computerized Adaptive Testing.

To illustrate the adaptive procedure, Figure 2 shows the trajectory of one CAT administration in a 46-year-old male participant with prevalent hostility and anxiety symptoms. The algorithm administered six items: from the demoralization subscale (*“I feel that I am unable to help myself”*; *“My life seems meaningless”*), hostility (*“I easily lose my temper”*; *“I feel easily irritable”*), depression (*“Little interest or pleasure in doing things”*), and anxiety (*“I feel nervous, tense, or restless”*). After each response, the latent trait estimates for demoralization, anxiety, hostility, and depression are updated, and their standard errors progressively decrease. Final estimates for this case converge to θ = 0.22 (demoralization), θ = 0.93 (anxiety), θ = 1.58 (hostility), and θ = 0.12 (depression), with corresponding SEs between 0.33 and 0.43, indicating precise recovery with only six items.

**Figure 3:**
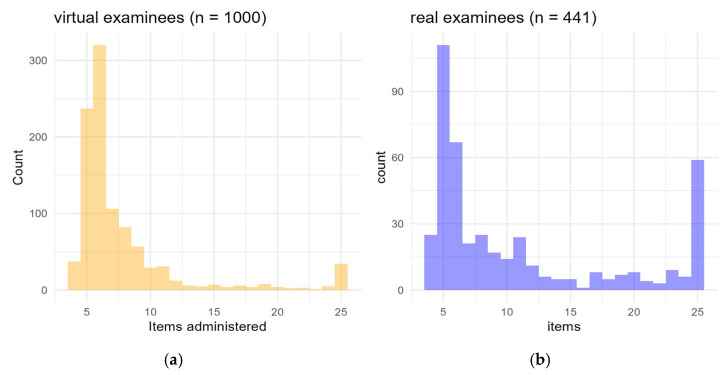
Distribution of administered items in CAT simulations (**a**) with virtual and real examinees (**b**).

We evaluated the CAT engine in simulated data with 1000 virtual respondents. Here, it required on average 7.76 items per simulated respondent (median = 6; IQR = 3; range = 4–25). The resulting distribution of administered items is shown in Figure 3a’s panel. Correlations between CAT-estimated and “true” latent trait values ranged from 0.94 to 0.97, with root mean square deviations of 0.24–0.28, indicating excellent recovery of trait estimate values and substantial test length reduction. The subsequent CAT simulation was applied to the original dataset of 441 real participants: it administered a mean of 10.95 items (median 7, IQR 10, range 4–25; Figure 3b panel). Accuracy for theta estimates from the four-factor model was very high across all factors, with correlations ranging from 0.948 to 0.953 and RMSD values between 0.269 and 0.298.

### 3.4. Development of the Web-Based Screening Tool

**Figure 4:**
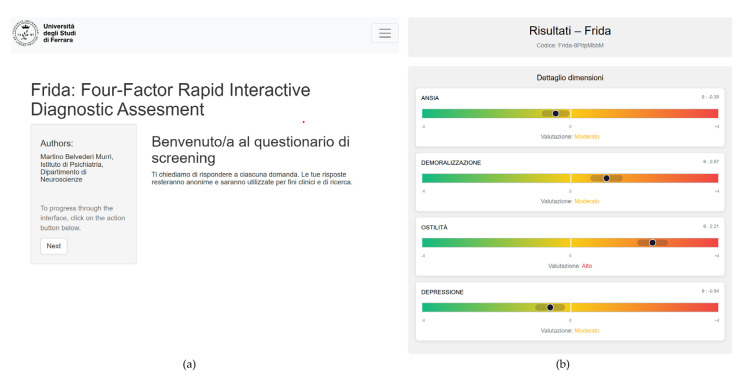
User interface of the FRIDA Platform (Italian version). (**a**) Initial access page. (**b**) Results interface displaying dimensional feedback (Anxiety, Demoralization, Hostility, Depression).

For clinical application, we implemented the CAT model into FRIDA (available at https://dk.aclai.unife.it/Frida (accessed on 08 September 2025). FRIDA is a web-based interface that provides a graphical display of estimated trait levels along a continuum, ranging from green (low risk) to red (high risk), with a visual indicator highlighting the respondent’s position. A warning signal is automatically triggered when the estimated theta exceeds the value of 0, corresponding to the upper half of the standard normal distribution. For demoralization, criterion validity was evaluated against the DCPR diagnosis as the gold standard. Using theta = 0 as the threshold, the tool demonstrated good discriminative ability (AUC = 0.816), with sensitivity of 80% and specificity of 67.5%. Negative predictive value was high (83.4%), while positive predictive value was lower (62.3%), indicating that the instrument is particularly effective at ruling out cases of demoralization (Figure 4).

## 4. Discussion

To our knowledge, this study is the first freely accessible, web-based CAT instrument for the rapid screening of psychopathology among hospital patients. The tool detects demoralization, anxiety, irritability, and depression—four domains in this patient population that are extremely common and debilitating.

Early identification of psychopathology in hospital patients is crucial, as timely recognition and intervention may improve outcomes and reduce morbidity. CLP has proven particularly effective when detection is early and systematic [21]. However, capturing the breadth of clinically relevant psychopathology often requires administering several instruments, each targeting a different domain [40]. This multiplied response burden may reduce feasibility in busy hospital settings, and different score computation may complicate the interpretation of results by clinicians. Adaptive Testing directly addresses these barriers by tailoring item selection to each respondent, improving patient experience, shortening test length, and maintaining high measurement precision across the severity spectrum [23,41].

This study follows and extends prior work on CAT in psychiatry and general medicine, where most applications have focused on unidimensional constructs, such as depression or anxiety, with CATs developed from PROMIS or fixed instruments [26,27]. In hospital or illness-specific settings, CATs have been successfully applied to improve the detection of targeted symptoms or domains, for example, substance use [25], negative mood in orthopaedic pain [27], or preoperative anxiety and depression [42], whereas multidimensional models have been mostly implemented in broader proprietary adaptive systems such as CAT-MH [24,43,44] or in large-scale assessments of cognition, daily activity, and mobility in older adults [28]. In contrast, FRIDA applies a multidimensional IRT framework to four correlated symptom domains—demoralization, anxiety, irritability, and depression—within a freely accessible tool designed specifically for hospital patients. CAT based on multidimensional IRT has been indicated as particularly efficient as it exploits the correlations among symptom domains, further reducing burden, preserving measurement precision while yielding separate yet interconnected estimates for complex traits, such as demoralization, anxiety, irritability, and depression [24].

The present tool addresses a practical clinical need: rapid, reliable screening in settings where patient time and attention are limited. By leveraging Item Response Theory, the system tailors administration to each respondent, presenting only the most informative items to achieve a predefined level of measurement precision. This allows for the combination of qualitative clinical insight with quantitative psychometric evidence, producing structured outputs that can be directly integrated into clinical decision making. In practice, this can facilitate early detection of distress across multiple domains, inform prioritization of interventions, and reduce assessment burden for both patients and staff.

Key strengths include the multidimensional design, which captures a broad spectrum of emotional distress; the adaptability of the CAT engine, which can be modified for different patient populations or clinical priorities; and its free, web-based availability, which enables wide dissemination. Limitations include the lack of screening of manifestations of severe mental disorders (e.g., bipolar disorder or psychoses). The reliance on an initial calibration sample from a single healthcare setting may affect generalizability and the lack of external validation against an interview-based criterion for measures other than demoralization. Another limitation is that the instruments differed in their assessment time frames (seven days for anxiety and irritability, two weeks for depression and demoralization, and one month for the DCPR diagnosis), which may have introduced some heterogeneity in symptom reporting. In addition, while the tool optimizes measurement efficiency, its performance in patients with cognitive impairment, language barriers, or very severe symptoms requires further evaluation. Moreover, the possibility of social desirability and recall bias should be acknowledged.

Future developments could address these issues by adapting the tool for specific populations, for example, through simplified language items, multilingual versions, or the possibility of proxy administration by caregivers or healthcare staff. Adaptation for patients with cognitive impairment could also draw on strategies explored in digital cognitive testing platforms [45], which emphasize usability through simplified instructions and accessibility features.

## 5. Conclusions

This study reports on the development of FRIDA, a freely available, multidimensional, web-based CAT for the rapid screening of the main four dimensions of psychological distress in hospital patients. The instrument offers a scalable, psychometrically robust solution for integrating mental health assessment into routine clinical workflows, with potential to improve early detection, documentation, and follow-up of psychiatric symptoms in general medical settings.

## Data Availability

The source code for FRIDA can be obtained from the corresponding author upon reasonable request for academic purposes. Due to clinical data protection policies, the dataset analysed in this pilot is not publicly accessible but may be made available in anonymized form upon approval by the relevant ethics committee. The copyright application for the FRIDA software (Version 0.1) has been filed and is currently under review.

## Supplementary Materials

The following supporting information can be downloaded at: https://www.mdpi.com/article/10.3390/jcm14196992/s1, Table S1: Sample characteristics, Table S2: Model loadings and parameters.

## Abbreviations

The following abbreviations are used in this manuscript:

AUC	Area Under the Curve
BSI-18	Brief Symptom Inventory-18
BSI-53	Brief Symptom Inventory-53
CAT	Computerized Adaptive Testing
CAT-MH	Computerized Adaptive Test for Mental Health
CFI	Comparative Fit Index
CLP	Consultation Liaison Psychiatry
DCPR	Diagnostic Criteria for Psychosomatic Research
DCPR/D	Diagnostic Criteria for Psychosomatic Research—Demoralization module
DSM-IV	Diagnostic and Statistical Manual of Mental Disorders, Fourth Edition
DS-24	Demoralization Scale, 24 items
FRIDA	Four-factor Rapid Interactive Diagnostic Assessment
GRM	Graded Response Model
IRT	Item Response Theory
KL	Kullback–Leibler divergence
PHQ-9	Patient Health Questionnaire-9
PROMIS	Patient-Reported Outcomes Measurement Information System
QMCEM	Quasi Monte Carlo Expectation-Maximization
ROC	Receiver Operating Characteristic
RMSEA	Root Mean Square Error of Approximation
RMSD	Root Mean Square Deviation
SE	Standard Error
SRMSR	Standardized Root Mean Square Residual
TLI	Tucker–Lewis Index
