# Supplementary material for: FRIDA: A Four-Factor Adaptive Screening Tool for Demoralization, Anxiety, Irritability, and Depression in Hospital Patients
Source: J Clin Med. 2025 Oct 2;14(19):6992. doi: 10.3390/jcm14196992 (PMC12524418; doi:10.3390/jcm14196992)
Supplement: Supplementary file 1 [file jcm-14-06992-s001.zip › jcm-3897713-supplementary.pdf]

Journal of
Clinical Medicine

MDPI

Supplementary Material

Article

FRIDA: A Four-Factor Adaptive Screening Tool for Demoralization, Anxiety, Irritability, and Depression in Hospital Patients

Table S1. Sample characteristics

Characteristic	Overall<br>N = 472[1]	Male<br>N = 212[1]	Female<br>N = 260[1]
**Age**	62.9 ± 17.7	64.7 ± 17.6	61.5 ± 17.7
Unknown	2	1	1
**Living arrangement**			
Alone	123 (26%)	37 (17%)	86 (33%)
Spouse/partner	262 (56%)	139 (66%)	123 (47%)
Family of origin	53 (11%)	24 (11%)	29 (11%)
Friends/roommates	13 (2.8%)	2 (0.9%)	11 (4.2%)
Other	21 (4.4%)	10 (4.7%)	11 (4.2%)
**marital_status**			
Single	80 (17%)	41 (19%)	39 (15%)
Married/cohabiting	265 (56%)	138 (65%)	127 (49%)
Separated/divorced	23 (4.9%)	11 (5.2%)	12 (4.6%)
Widowed	104 (22%)	22 (10%)	82 (32%)
**education**			
No qualification	32 (6.8%)	6 (2.8%)	26 (10%)
Primary school	164 (35%)	70 (33%)	94 (36%)
Middle school	134 (28%)	65 (31%)	69 (27%)
High school diploma	106 (22%)	54 (25%)	52 (20%)
University degree	36 (7.6%)	17 (8.0%)	19 (7.3%)
**Admission_reason**			
Dermatologic	0 (0%)	0 (0%)	0 (0%)
Gastroenterologic	80 (17%)	38 (18%)	42 (16%)
Suicide attempt	19 (4.0%)	7 (3.3%)	12 (4.6%)
Cardio/vascular	83 (18%)	41 (19%)	42 (16%)
Onco/hematologic	42 (8.9%)	23 (11%)	19 (7.3%)
Respiratory	48 (10%)	28 (13%)	20 (7.7%)
Neurologic	52 (11%)	19 (9.0%)	33 (13%)
Psychiatric	0 (0%)	0 (0%)	0 (0%)
Infectious disease	57 (12%)	34 (16%)	23 (8.9%)
Rheumatologic	15 (3.2%)	2 (0.9%)	13 (5.0%)
Nephrologic	4 (0.8%)	3 (1.4%)	1 (0.4%)
Diabetologic	4 (0.8%)	1 (0.5%)	3 (1.2%)
Aspecific symptoms	17 (3.6%)	3 (1.4%)	14 (5.4%)

Journal of Clinical Medicine

MDPI

Characteristic	Overall<br>N = 472[1]	Male<br>N = 212[1]	Female<br>N = 260[1]
Other	50 (11%)	13 (6.1%)	37 (14%)
Unknown	1	0	1
**work_status**			
Unemployed	29 (6.2%)	12 (5.7%)	17 (6.5%)
Clerk	29 (6.2%)	13 (6.2%)	16 (6.2%)
Worker	35 (7.4%)	20 (9.5%)	15 (5.8%)
Self-employed	24 (5.1%)	15 (7.1%)	9 (3.5%)
Housewife	31 (6.6%)	0 (0%)	31 (12%)
Student	13 (2.8%)	4 (1.9%)	9 (3.5%)
Retired	281 (60%)	140 (66%)	141 (54%)
Other	29 (6.2%)	7 (3.3%)	22 (8.5%)
Unknown	1	1	0

[1] Mean ± SD; n (%)

Journal of Clinical Medicine

MDPI

Table S2. Model loadings and parameters

Item	F1 demoralization	F2 anxiety	F3 hostility	F4 depression	h2	a1	a2	a3	a4	b1	b2	b3	b4
demo1	0.503				0.253	0.99				-1.391	0.475	1.744	2.972
demo2	0.879				0.773	3.142				0.117	0.523	1.229	2.052
demo3	0.800				0.640	2.268				0.054	0.540	1.257	2.127
demo4	0.776				0.603	2.096				-0.067	0.487	1.068	1.784
demo5	0.653				0.427	1.468				-0.358	0.404	1.364	2.943
demo6	0.749				0.561	1.926				-1.860	-0.285	0.754	1.69
demo7	0.654				0.427	1.47				0.080	0.665	1.524	2.476
demo8	0.681				0.464	1.583				-0.729	0.036	0.930	1.796
demo9	0.871				0.759	3.021				-0.006	0.400	0.968	1.671
demo10	0.371				0.138	0.68				0.700	1.363	2.858	4.807
demo11	0.497				0.247	0.976				-0.711	0.380	1.601	3.633
demo12	0.765				0.585	2.021				-1.836	-0.098	1.169	2.207
demo13	0.356				0.126	0.647				-0.507	0.648	1.983	4.481
demo14	0.858				0.736	2.843				0.640	1.026	1.474	2.131
demo15	0.499				0.249	0.981				-1.030	0.306	1.356	3.027
demo16	0.600				0.360	1.276				-0.716	0.185	1.073	2.508
demo17	0.413				0.170	0.771				-0.669	1.408	3.380	4.886
demo18	0.728				0.529	1.805				-1.400	-0.588	0.200	1.104
demo19	0.497				0.247	0.975				-1.517	0.611	2.354	4.511
demo20	0.857				0.734	2.83				0.859	1.204	1.636	2.174
demo21	0.856				0.733	2.821				-0.717	-0.018	0.717	1.491
demo22	0.837				0.700	2.601				-0.622	-0.099	0.603	1.389
demo23	0.73				0.532	1.816				0.258	0.697	1.218	1.887
demo24	0.778				0.605	2.105				-0.790	-0.256	0.371	1.132
bsi3		0.815			0.664		2.392			-0.841	-0.244	0.397	1.243
bsi6		0.841			0.708		2.651			-0.573	-0.004	0.531	1.32

Journal of Clinical Medicine

MDPI

Item	F1 demoralization	F2 anxiety	F3 hostility	F4 depression	h2	a1	a2	a3	a4	b1	b2	b3	b4
bsi9		0.779			0.607		2.116			0.558	0.951	1.385	2.091
bsi12		0.790			0.624		2.193			0.741	1.202	1.615	2.256
bsi15		0.753			0.567		1.948			0.379	0.782	1.288	2.354
bsi18		0.727			0.528		1.802			-0.065	0.502	1.148	2.018
bsi6x			0.839		0.704			2.626		-0.129	0.473	1.033	1.787
bsi13x			0.832		0.692			2.554		0.605	1.079	1.638	2.35
bsi40			0.912		0.832			3.786		1.364	1.697	1.932	2.473
bsi41			0.902		0.813			3.548		1.222	1.661	1.874	2.491
bsi46			0.757		0.573			1.971		0.755	1.333	2.019	2.956
phqa				0.783	0.613				2.141	-0.268	0.478	1.146	
phqb				0.876	0.767				3.09	-0.502	0.269	0.896	
phqc				0.493	0.243				0.964	-0.611	0.606	1.578	
phqd				0.66	0.435				1.494	-1.614	-0.446	0.520	
phqe				0.468	0.219				0.902	-0.043	0.916	1.838	
phqf				0.762	0.580				2.002	0.432	1.087	1.787	
phqg				0.617	0.381				1.335	0.329	1.319	2.191	
phqh				0.539	0.291				1.089	1.383	2.322	3.422	
phqi				0.862	0.743				2.891	0.970	1.580	2.031	
